# Altered Cortico-Striatal Connectivity in Offspring of Schizophrenia Patients Relative to Offspring of Bipolar Patients and Controls

**DOI:** 10.1371/journal.pone.0148045

**Published:** 2016-02-17

**Authors:** Cristina Solé-Padullés, Josefina Castro-Fornieles, Elena de la Serna, Soledad Romero, Anna Calvo, Vanessa Sánchez-Gistau, Marta Padrós-Fornieles, Inmaculada Baeza, Núria Bargalló, Sophia Frangou, Gisela Sugranyes

**Affiliations:** 1 August Pi i Sunyer Biomedical Research Institute (IDIBAPS), Barcelona, Spain; 2 Department of Child and Adolescent Psychiatry and Psychology, SGR489, Institute of Neuroscience, Hospital Clínic of Barcelona, Barcelona, Spain; 3 Department of Psychiatry and Clinical Psychobiology, University of Barcelona, Barcelona, Spain; 4 Biomedical Research Networking Centre Consortium (CIBERSAM), Barcelona, Spain; 5 Magnetic Resonance Imaging Core facility, Hospital Clinic of Barcelona, Barcelona, Spain; 6 Bioengineering, Biomaterials and Nanomedicine (CIBER-BBN), GIB-UB, Barcelona, Spain; 7 Centre for Diagnostic Imaging (CDI), Hospital Clinic of Barcelona, Barcelona, Spain; 8 Department of Psychiatry, Icahn School of Medicine at Mount Sinai, New York City, United States of America; Mayo Clinic, UNITED STATES

## Abstract

Schizophrenia (SZ) and bipolar disorder (BD) share clinical features, genetic risk factors and neuroimaging abnormalities. There is evidence of disrupted connectivity in resting state networks in patients with SZ and BD and their unaffected relatives. Resting state networks are known to undergo reorganization during youth coinciding with the period of increased incidence for both disorders. We therefore focused on characterizing resting state network connectivity in youth at familial risk for SZ or BD to identify alterations arising during this period. We measured resting-state functional connectivity in a sample of 106 youth, aged 7–19 years, comprising offspring of patients with SZ (*N* = 27), offspring of patients with BD (*N* = 39) and offspring of community control parents (*N* = 40). We used Independent Component Analysis to assess functional connectivity within the default mode, executive control, salience and basal ganglia networks and define their relationship to grey matter volume, clinical and cognitive measures. There was no difference in connectivity within any of the networks examined between offspring of patients with BD and offspring of community controls. In contrast, offspring of patients with SZ showed reduced connectivity within the left basal ganglia network compared to control offspring, and they showed a positive correlation between connectivity in this network and grey matter volume in the left caudate. Our findings suggest that dysconnectivity in the basal ganglia network is a robust correlate of familial risk for SZ and can be detected during childhood and adolescence.

## Introduction

Schizophrenia (SZ) and bipolar disorder (BD) share genetic risk factors [1, 2], clinical symptoms [3], cognitive deficits [4], and brain structural and functional alterations [1, 5–7].

Resting-state functional magnetic resonance imaging (rs-fMRI) has enabled the study of the brain’s intrinsic functional architecture [8]. In the present study we focus on resting-state networks (RSN) that are reliably identified across samples and are also implicated in both disorders [9–12, 7].

The default mode network (DMN) comprises the medial prefrontal cortex, posterior cingulate/precuneus, lateral temporal cortex and inferior parietal lobule [13], and is deactivated during cognitive processes requiring externally oriented attention [14]. Aberrant connectivity within the DMN has been observed in adult [15] and adolescent SZ patients [16]. Studies in adult BD have reported conflicting results with either no abnormalities [17] or hypo-connectivity in the DMN [18]. The executive control network (ECN), which includes the dorsolateral prefrontal and posterior parietal cortices [19], is implicated in planning, decision making and sustained attention [20]. Reduced ECN connectivity has been observed in adult SZ patients [21], while hyperconnectivity has been reported in paediatric BD [22]. The salience network (SN) includes the fronto-insular cortex and the dorsal anterior cingulate, and has a role in emotional processing [19]. Studies in adult SZ have shown hypoconnectivity within this network [15], while it may be increased in BD [17]. In the basal ganglia network (BGN), which includes the striatum, inferior frontal gyri and thalami, similarly reduced connectivity has been reported in both SZ and BD [23, 24].

Connectivity within the DMN has been found to be increased in adult unaffected SZ relatives [25–27] and decreased in BD relatives [28]. For the ECN, a recent report found reduced connectivity within the right inferior frontal gyrus in young adult offspring of patients with a psychotic spectrum disorder [29] while the only study in young offspring of BD patients so far revealed increased connectivity in the ventrolateral prefrontal cortex [30]. A recent study in adult relatives of patients with first episode psychosis has also reported hypoconnectivity within the BGN [31]. To our knowledge the Bipolar and Schizophrenia Network on Intermediate Phenotypes study is the only study to date to compare RSN between adult relatives of SZ or BD comparatively [28, 11, 24], and has reported reduced connectivity within the frontal/thalamic/basal ganglia network in relatives of SZ or BD probands, with a more prominent decrease in striatal connectivity for SZ relatives.

The evidence summarized above indicates that abnormal functional connectivity in SZ and BD may be related to familial risk. RSN undergo substantial reorganization throughout childhood and adolescence [32], during which there is a shift in the balance between prefrontal and subcortical connectivity [33]. The engagement of the prefrontal cortex approaches adult levels early in adolescence [34] while striatal networks experience significant maturation throughout adolescence and into adulthood [35]. Studies employing neuropsychological measures have revealed that child and adolescent offspring of patients with SZ (SzO) or BD (BpO) share difficulties in verbal and visual episodic memory, executive function and intelligence [36, 37], although group specific impairment has also been described [37]. However, no study so far has examined intrinsic connectivity of resting state networks implicated in cognition in young offspring of patients with SZ or BD.

In this context, we set out to test whether intrinsic functional connectivity of cortical and cortico-subcortical networks occurring during childhood and adolescence are disrupted by familial risk of SZ or BD. We hypothesized that young offspring of SZ or BD patients would show abnormal intrinsic connectivity in regions where dysconnectivity has been reported in adult patients or relatives of SZ and BD, namely frontal and frontostriatal areas. To address this hypothesis we used Independent Component Analysis (ICA) to identify the DMN, ECN, SN and BGN in a sample of child and adolescent offspring. Our further aim was to examine the relationship between resting state connectivity differences and grey matter volume, cognitive and clinical measures.

## Materials and Methods

This study has been approved by the Comitè Ètic d'Investigció Clínica (CEIC), Hospital Clínic, Barcelona, Spain. For participants aged under 18, parents provided written informed consent and participants older than 12 provided assent. Participants aged 18 and over gave written informed consent. Families received compensation for their time and travel expenses.

### Subjects

This study was undertaken at the Child and Adolescent Psychiatry and Psychology Department of the Hospital Clinic of Barcelona, Spain.

Psychiatrists of adult inpatient and outpatient units were asked to identify BD and SZ patients who had children aged 7 to 19 years. Exclusion criteria for index parents were: intellectual disability, and drug or medically-induced psychosis or mania. 58 families (31 BD and 27 SZ) were assessed. Community control parents were recruited through advertisements posted in the community within the same geographical area as patients. Exclusion criteria for community control parents were: intellectual disability, severe neurological conditions, and personal or 1st degree family history of SZ or BD spectrum disorders. In order to reduce selection bias, parents who stated to be specifically motivated to participate due to concerns about school performance or emotional or behavioural problems in their offspring were also excluded. 32 control families met inclusion criteria.

Exclusion criteria for all offspring included: intellectual disability according to the Diagnostic and Statistical Manual of Mental Disorders, Fourth Edition (American Psychiatric Association, APA, 1994, [38]) criteria (IQ below 70 and impaired functioning), history of head injury with loss of consciousness or severe neurological conditions. Additionally, CC-offspring had no personal, 1st or 2^nd^ degree family history of SZ or BD spectrum disorders. Thirty-three SZ-offspring, 47 BD-offspring and 46 offspring of community control parents (CC-offspring) were recruited. Further information on the recruitment of the sample is provided in Sánchez-Gistau and colleagues [39].

### Clinical/cognitive assessments

Clinical and cognitive assessments were carried out by experienced psychiatrists and psychologists at the Child and Adolescent Outpatient Department of the hospital. Parental and offspring interviews were conducted by different team members, each blind to the others’ assessment. Parental socioeconomic status (SES) was estimated using the Hollingshead Scale [40]; considering the highest SES among both parents. Clinical diagnoses were based on the Spanish version of the Structured Interview for DSM-IV disorders (SCID) [41] for parents and offspring aged 18 years or older. Offspring under 18 were assessed by child and adolescent psychiatrists using the Spanish version of The Schedule for Affective Disorders and Schizophrenia for School-Age Children—Present and Lifetime version (K-SADS–PL) [42] administered separately to parents and children. Details on past or current psychopharmacological treatments were also registered.

Symptoms were evaluated with the Scale of Prodromal Symptoms (SOPS) within the Structured Interview for Prodromal Symptoms [43], the Young Mania Rating Scale [44] and the Hamilton Depression Rating Scale [45]. Overall clinical severity was assessed with the Clinical Global Impression index [46] and functionality with the Global Assessment of Functioning scale from DSM-IV-TR. A measure of general cognitive capacity was estimated using the Vocabulary, Similarities, Block Design and Matrix Reasoning subtests of the Wechsler Intelligence Scale for Children (WISC-III) [47] or the Wechsler Adult Intelligence Scale (WAIS-III) [48], for participants aged sixteen and over. A more thorough neuropsychological assessment was also carried out to assess executive function (Wisconsin Card Sorting Test), attention (Continuous Performance Test; Digit Span, WAIS/WISC-III) and memory (Test of Memory and Learning: immediate and delayed recall).

### Imaging data acquisition

Scans were obtained on a 3 Tesla Siemens Magnetom Trio Tim (Siemens Medical Systems, Germany) magnetic resonance scanner at the Centre for Image Diagnosis, Hospital Clínic, Barcelona. Participants were instructed to remain as still as possible for the duration of the scanning session. Soft pads were placed at the sides of their heads in order to help avoid further movement. A high-resolution T1-weighted 3-dimensional (3D) magnetization-prepared rapid sequence was acquired with the following parameters: 240 sagital slices; TR = 2300 ms; TE = 3.01 ms; slice thickness = 1 mm; inversion time (TI) = 900; FOV = 394x240; matrix size = 256×256; and flip angle = 9°. An 8-minute rs-fMRI sequence was also acquired, prior to which participants were instructed to keep their eyes closed and not fall asleep. Acquisition parameters were as follows: 240 volumes, TR = 2000 ms; TE = 29 ms; matrix size = 480x480; slice thickness = 4 mm, acquisition matrix = 80x80 mm, 32 slices, voxel size 3x3x4 mm^3^.

Following acquisition, images were inspected to exclude participants with macroscopic abnormalities. One BD-offspring was excluded due to ventriculomegalia. rs-fMRI Images were also inspected for excessive head motion during pre-processing, defined as 1.5 mm translation and 1.5 degrees rotation in any of the x, y or z directions. This led to exclusion of 19 participants (6 SZ-offspring, 7 BD-offspring, 6 CC-offspring). The total sample analysed consisted of 27 SZ-offspring, 39 BD-offspring and 40 CC-offspring.

### Statistical analysis

Demographic, clinical and cognitive data were analysed with the Statistical Package for Social Sciences (SPSS v.20) employing repeated measures analyses of variance and chi-square tests. Non-parametric testing and log-transformation of variables were undertaken when assumption of normality was not met (Kolmogorov-Smirnov). Age correlated with performance on immediate and delayed memory tasks as well as with digit span, therefore cognitive measures were regressed for age prior to performing between-group comparisons.

### Image processing and analysis

rs-fMRI data was preprocessed with the Statistical Parametric Mapping software (SPM8), running in Matlab (R2013a), as follows: reorientation of 3D and fMRI images along the anterior and posterior commissural line; realignment with motion correction and inspection of outputs; corregistration of anatomical to functional data; normalization into standard Montreal Neurological Institute (MNI) space [49] and smoothing with an 8 mm FWHM kernel. Images were then subjected to a single spatial ICA, employing the Group ICA fMRI Toolbox (GIFT v3.0a), performed in 3 stages: First, principal component analyses (PCA) was conducted twice: the dataset was first reduced to 30 components, and then was further reduced to 20 components. The first PCA reduction step is at the subject level and the second one at the group level. Subject-level PCA allows preservation of differences between subjects at the same time that it emphasizes the similarities between subjects by projecting data into a common space. This point enables acquisition of mean data for the subjects and is required to make the data computationally tractable. On the other hand, by conducting group-level PCA, data is further reduced into a set number of components and independent group spatial maps [50]. Second, the Infomax algorithm was used to decompose the reduced dataset into maximally independent component images; and finally, back-reconstruction of components using the Group ICA tool. Components were sorted by spatial correlation with a template of the DMN provided by the GIFT software, as well as with templates for the ECN, BGN, and anterior SN available at http://findlab.stanford.edu/functional_ROIs.html [51]. Components showing the greatest correlation with the templates were exported to SPM8, where one sample t-tests were carried out to determine anatomical regions within each network. Then, ANCOVA was conducted to test the effect of group membership on within network connectivity, with age and gender as covariates. A grey matter mask was obtained from the smoothed, modulated and normalised grey matter images for the whole sample, which was used as an inclusive mask for all second-level analyses. Significant main effects of group were followed-up with pairwise comparisons. Results were interpreted at a voxel-wise threshold of p<0.001 uncorrected and a cluster-wise threshold of p<0.05 FWE corrected. Connectivity signal values from clusters showing significant group differences were extracted using a volume-of-interest (VOI) approach in order to examine the relationship between magnitude of connectivity and clinical, cognitive and grey matter volume (GMV) measures.

Computation of GMV within the clusters identified in the above comparisons was undertaken with a customised script running in Matlab. T1 images underwent a unified segmentation followed by smoothing with an 8mm FWHM kernel. In order to adjust for head size, a grey matter ratio was obtained for each subject (GMV of the cluster was divided by the subject’s total intracranial volume (TIV), [52]). TIV was computed using the Freesurfer pipeline [53]. ANCOVA was used to determine the effect of group on GMV within clusters identified in the above comparisons, with age and gender as covariates. The relationship between rs-fMRI signal (within the cluster showing between group differences) and clinical, cognitive and volumetric data was assessed using partial correlations controlling for age and gender.

Finally, analyses were repeated excluding offspring with a history of any axis I DSM-IV diagnosis. Confirmatory analyses were also performed to take into account the effect of having a sibling in the sample (5 SZ, 9 BD, and 8 CC families contributed more than one offspring to the study): mixed models were conducted with SPSS, where the mean connectivity values were included as dependent variable, ‘sibship’ as random effects, and age and gender as fixed effects.

## Results

Out of the final 22 SZ families, 18 parents had a diagnosis of SZ (DSM-IV codes 295.10–90), while 3 parents had a diagnosis of schizoaffective disorder (DSM-IV codes 295.40). Out of the 28 BD families, 18 parents had BD-I (DSM-IV codes 296.40–70), and 7 had BD-II (DSM-IV codes 296.89). All other current and lifetime psychiatric diagnoses of parents (proband parents, co-parents and community control parents) are shown in Table 1. No significant differences in terms of rates of lifetime or current axis I diagnoses were observed between the three groups of parents (SZ, BP, CC; *χ*² = 1.263, p = 0.532).

**Table 1:** Clinical characteristics of parents.

	Schizophrenia proband parents (*N* = 19)	Schizophrenia co-parents (*N* = 17)	Bipolar Disorder proband parents (*N* = 30)	Bipolar Disorder co-parents (*N* = 23)	Community Control parents (*N* = 53)
	Mean (standard deviation) / *N* (%)				
**Age**	41.69 (6.16)	46.61 (9.75)	45.14 (5.52)	45.87 (5.51)	45.09 (5.41)
Total lifetime axis I disorders*	7 (36.84%)	10 (58.82%)	11 (36.67%)	9 (39.13%)	19 (35.86%)
**Lifetime attention deficit hyperactivity disorders**	0	0	1 (3.33%)	0	0
**Lifetime mood disorders**	2 (10.53%)	4 (23.53%)	5 (16.67%)	2 (8.70%)	3 (5.66%)
**Lifetime anxiety disoders**	1 (5.26%)	0	3 (10%)	0	3 (5.66%)
**Lifetime adjustment disorders**	2 (10.53%)	6 (35.29%)	2 (6.67%)	4 (17.39%)	11 (20.75%)
**Lifetime substance-related disorders**	1 (5.26%)	0	0	2 (8.70%)	0
**Lifetime eating disorders**	1 (5.26%)	0	0	1 (4.35%)	2 (3.77%)
Total current axis I disorders*	0	6 (35.29%)	6 (20%)	7 (30.43)	9 (16.98%)
**Current attention deficit hyperactivity disorders**	0	0	2 (6.67%)	0	0
**Current mood disorders**	0	3 (17.65%)	0	0	0
**Current anxiety disoders**	0	2 (11.76%)	2 (6.67%)	4 (17.39%)	3 (5.66%)
**Current adjustment disorders**	0	1 (5.88%)	1 (3.33%)	2 (8.70%)	6 (11.32%)
**Current substance-related disorders**	0	0	1 (3.33%)	1 (4.35%)	0

* All axis I-related disorders other than schizophrenia and bipolar disorder are considered.

Demographic, clinical, cognitive and neuroimaging data of the offspring are depicted in Table 2. There was no significant effect of group on age, sex or TIV. SZ-offspring had lower parental SES relative to the other groups. SZ had greater lifetime prevalence of attention deficit hyperactivity disorder (ADHD) than CC-offspring. There were no other clinical differences, except for SZ and BD-offspring showing higher SOPS negative scores than CC-offspring. Six participants (5 SZ-offspring and 1 BD-offspring) were receiving treatment with psychostimulants for treatment of ADHD at the time of scanning; no other participant had received any other form of psychiatric medication. CC-offspring had higher IQ relative to SZ-offspring. No significant differences were seen in any other cognitive domain. No differences in peak translation or rotation motion parameters were observed between groups.

**Table 2:** Socio-demographic, clinical and cognitive characteristics of the sample.

	Schizophrenia Offspring (*N* = 27)	Bipolar Disorder Offspring (*N* = 39)	Community Control Offspring (*N* = 40)			
	Mean (standard deviation) / *N* (%)			Statistic	P value	Significant pair-wise comparisons, Bonferroni corrected p (0.05/3 = 0.017)
**Age**	11.96 (3.41)	13.87 (3.49)	13.45 (3.76)	4.21^a^	0.122	
**Sex** (female**)**	13 (48.15%)	22 (56.41%)	24 (60%)	0.932^b^	0.628	
**Socio-economic status**^1^	38.56 (12.17)	47.05 (13.11)	50.23 (10.86)	12.05^a^	0.002	CC-offspring>SZ-offspring p<0.0001 BP-offspring>SZ-offspring p = 0.014
**Lifetime attention deficit hyperactivity disorders**	9 (33.33%)	6 (15.38%)	1 (2.5%)	11.962^b^	0.003	SZ-offspring > CC-offspring (p<0.0001);
**Lifetime mood disorders**^2^	2 (7.41%)	1 (2.56%)	2 (5%)	0.844^b^	0.656	
**Lifetime anxiety disorders**^3^	2 (7.41%)	5 (12.82%)	0	5.30^b^	0.071	
**Lifetime disruptive behaviour disorders**^4^	1 (3.70%)	1 (2.56%)	1 (2.5%)	0.101^b^	0.951	
**YMRS**^5^	2.08 (3.43)	1.32 (1.85)	0.94 (1.72)	1.555^a^	0.459	
**HDRS**^6^	0.8 (1.22)	2.23 (4.33)	0.50 (1.25)	3.422^a^	0.181	
**SOPS-P**^7^	1.36 (2.18)	1.10 (2.60)	0.61 (1.57)	2.824^a^	0.244	
**SOPS-N**^8^	1.00 (1.57)	1.61 (3.40)	0.31 (1.06)	8.185^a^	0.017	BD-offspring > CC-offspring (p = 0.016); SZ-offspring > CC-offspring (p = 0.006)
**Maximum translation movement (mm)**	0.62 (0.38)	0.61 (0.38)	0.53 (0.27)	0.779^a^	0.678	
**Maximum rotation movement (degrees)**	0.57 (0.44)	0.50 (0.36)	0.45 (0.34)	1.097^a^	0.578	
**Total intracranial volume** (cc)	1501.03 (153.79)	1541.12 (143.64)	1529.49 (168.45)	2.176^a^	0.337	
**Total Grey matter volume** (cc)	715.14 (69.50)	713.39 (58.19)	718.43 (72.02)	0.057^c^	0.944	
**General Cognitive Capacity**^9^	101.30 (14.77)	108.26 (11.80)	110.60 (12.32)	4.40^c^	0.015	CC-offspring > SZ-offspring (p = 0.013)
**WCST**^10^**-correct answers**	70.14 (7.61)	68.38 (9.77)	71.67 (9.13)	0.863^c^	0.426	
**WCST-complete categories**	5.33 (1.68)	5.96 (0.20)	5.84 (0.50)	2.724^c^	0.072	
**TOMAL**^11^**-immediate recall**	76.09 (8.78)	81.85 (8.66)	80.67 (8.24)	0.7^c^	0.5	
**TOMAL-delayed recall**	9.86 (2.01)	10.61 (1.50)	10.59 (1.46)	0.623^c^	0.539	
**CPT**^12^**-reaction time**	51.23 (9.66)	51.57 (11.65)	46.41 (9.20)	2.566^c^	0.083	
**Digit span**	14.05 (2.73)	16.46 (3.31)	16.03 (2.80)	2.186 ^c^	0.119	

^1^Hollingshead Index.

^2^Mood disorders: dysthymia and adjustment disorder with depressed mood.

^3^Anxiety disorders: simple phobia, generalized anxiety disorder and obsessive-compulsive disorder.

^4^Disruptive behaviour disorders: oppositional defiant and conduct disorder.

^5^Young Mania Rating Scale;

^6^Hamilton Depression Rating Scale;

^7^Scale of Prodromal Symptoms: positive symptoms;

^8^Scale of Prodromal Symptoms: negative symptoms;

^9^Wechsler Intelligence Scale for Children-III/Wechsler Adult Intelligence Scale-III

^10^Wisconsin Card Sorting test,

^11^Test of Memory And Learning,

^12^Continuous Performance Test.

^a^Kruskal-Wallis;

^b^Chi-square;

^c^ANOVA. Pairwise comparisons were corrected for Bonferroni, setting the p value threshold at 0.017 (alpha value divided into three main comparisons).

### Independent component analysis

Spatial maps for each network are described in Table 3.

**Table 3:** Resting-state components.

Resting State Networks	Correlation (*r*) with spatial template for the whole sample	Correlation (*r*) with spatial template for offspring without a history of psychiatric disorders	Main anatomical locations
**Default Mode Network**	0.59351	0.52258	Left: Precentral and paracentral lobule, superior frontal, inferior occipital cortices, inferior parietal lobe and posterior cingulate. Right: Middle/inferior/superior frontal cortex, precuneus, middle occipital, inferior/superior parietal and middle temporal cortices.
**Basal Ganglia Network**	0.4039	0.41902	Pallidum, anterior cingulate, thalami, putamen, caudate, right posterior cingulate, right superior frontal and right middle temporal cortices.
**Left Executive Control Network**	0.42065	0.48866	Left: orbitofrontal, superior/inferior/middle/medial frontal, inferior parietal and inferior temporal cortices. Right: supramarginal gyrus and middle/superior occipital cortex.
**Right Executive Control Network**	0.4239	0.42226	Left: precuneus, paracentral lobule and posterior cingulate. Right: superior/middle/inferior/medial frontal, precentral, inferior parietal and middle temporal cortices, fusiform gyrus, precuneus, and insula.
**Anterior Salience Network**	0.56265	0.39225	Left: precentral cortex, amygdala, anterior/middle cingulate, paracentral lobule, medial/superior/middle orbital frontal cortex, and globus pallidum. Right: superior/middle/inferior frontal cortices, cerebellum, inferior temporal cortex and inferior parietal lobe.

**Fig 1:**
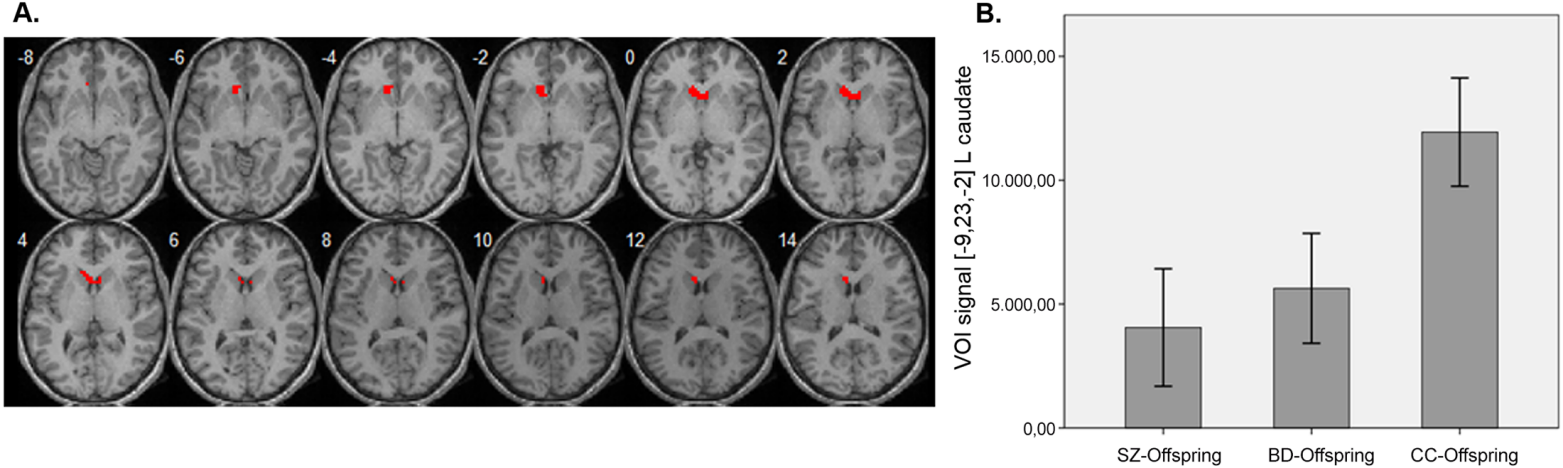
A. Cluster showing group differences within the Basal Ganglia Network overlaid on a normalised 3D image. B. Mean signal intensity for each group within the global maxima in the BGN. Error bars represent the 95% confidence interval.

An effect of group membership was detected for the BGN (F = 13.07, p_FWE_ = 0.019, *k* = 55, MNI coordinates x,y,z [−9,23,−2]). Pairwise analyses showed that this finding was driven by the SZ-offspring versus CC-offspring comparison: SZ-offspring showed reduced connectivity in the left caudate nucleus and anterior cingulate, extending to the left olfactory cortex (t = 4.45, p_FWE_ = 0.012, *k* = 80, MNI [[−9,23,−2]). This pairwise comparison survived Bonferroni correction (p-value threshold 0.05/3 = 0.017). There were no differences between BD-offspring and CC-offspring, or between SZ-offspring and BD-offspring. Fig 1A illustrates the effect of group on connectivity within the BGN, including mean signal intensity at the left caudate (Fig 1B). When IQ was entered in the model, results remained unchanged (group effect, F = 10.97, p_FWE_ = 0.022, k = 53, MNI [[−9,23,1]; pairwise comparison SZ-offspring < CC-offspring t = 4.21, p_FWE_ = 0.004, k = 100, MNI [–3,17,1], left olfactory cortex extending to the caudate nuclei). A group effect was also detected in the right ECN, albeit at an uncorrected threshold (F = 10.96, p_uncorrected_ = 0.026, *k* = 23, MNI [36,–79,31]); this was driven by increased connectivity in BD-offspring relative to the other groups, particularly SZ-offspring (t = 4.68, p_FWE_ = 0.035, *k* = 61, MNI [36,–79,31]). No significant group differences were seen for the DMN or SN.

Given that volumetric reduction of the caudate nucleus has been associated with ADHD [54] and that treatment with psychostimulants may modulate caudate activity [55], we conducted a second-level analysis excluding 9 SZ-offspring, 6 BD-offspring and 1 CC-offspring with ADHD, among which were the six subjects receiving psychotropic medications at the time of scanning. Results remained near significant for the same cluster (t = 4.60, p_FWE_ = 0.05, *k* = 54, MNI [–9,26,–5]). Analyses excluding subjects with lifetime history of axis I disorders did not yield any significant changes to the group differences within the BGN, albeit at an uncorrected statistical threshold (SZ-offspring<CC-offspring: t = 4.82, p_unc_ = 0.012, *k* = 43, MNI [–9,26,–5]). Analyses taking into account the effect of sibship in this same region within the BGN confirmed the abovementioned results (F = 11.87, p<0.0001; Bonferroni correction).

### Relationship between RSN and brain structural, clinical and cognitive variables

**Fig 2:**
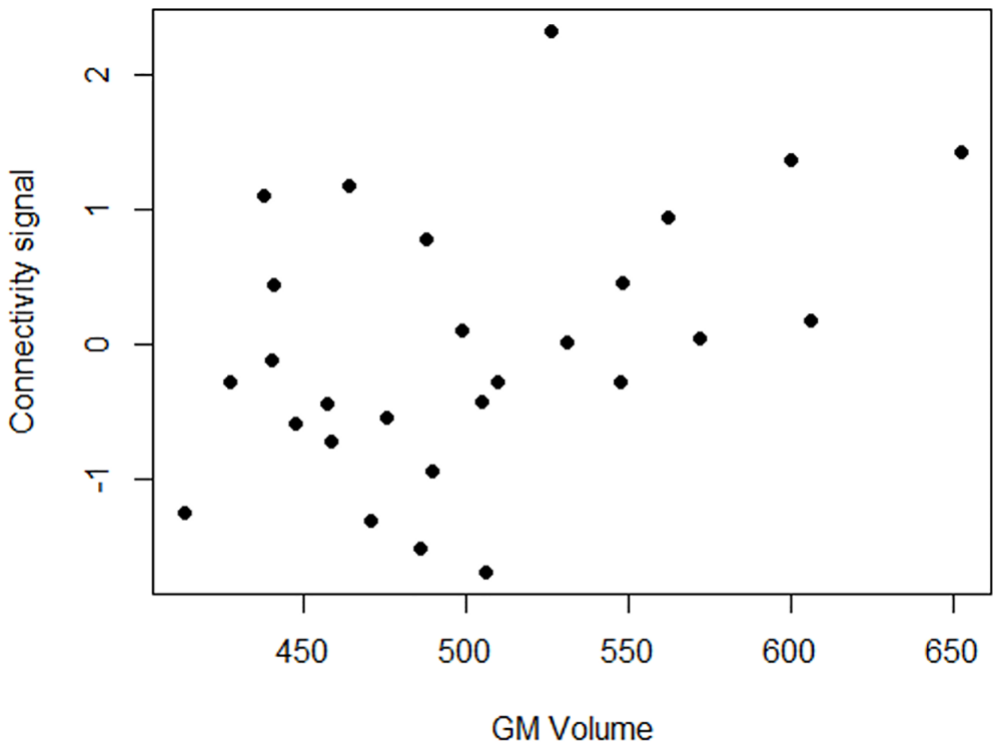
Partial regression plot depicting correlation between VOI signal values and grey matter volume in the left caudate (SZ-offspring).

GMV of the cluster showing significant between group differences in the BGN was measured for each participant. No differences in GMV/TIV ratio were seen between groups (F = 0.36, p = 0.699). Correlation analyses were also conducted applying Bonferroni correction (p = 0.05 divided by the number of structural, clinical and cognitive variables (12), yielded a threshold of statistical significance at p < 0.004). There was a positive correlation between rs-fMRI signal and regional GMV in SZ-offspring only (R^2^ = 0.598, p = 0.032, unstandardized coefficient ß = 37.54, standard error = 16.47, see Fig 2). No significant associations were observed between the rs-fMRI signal and clinical/cognitive variables.

## Discussion

This is, to the best of our knowledge, the first study to directly compare rs-fMRI in child and adolescent offspring of patients with SZ and BD. We found reduced left BGN connectivity in SZ-offspring relative to CC-offspring that correlated with GMV in the left caudate, and no differences in connectivity between BD-offspring and CC-offspring.

Hypoconnectivity in the left BGN in SZ-offspring involved the left caudate nucleus, the anterior cingulate and ventromedial prefrontal cortex. These findings remained, at an uncorrected threshold, after excluding offspring with pre-existing lifetime psychiatric diagnoses. Decreased resting-state corticostriatal connectivity has been previously reported in siblings and parents of patients with psychotic disorders [31, 11]. The present study expands these findings and demonstrates that BGN hypo-connectivity may be a robust correlate of familial risk for SZ, regardless of age. Unlike Fornito and colleagues, we did not confirm an association between BGN connectivity and clinical and cognitive symptoms. These authors found a correlation between fronto-striatal connectivity and positive and negative symptoms in patients with first-episode psychosis [31], but they failed to assess this relationship in their group of first degree relatives. Our sample involved young offspring of BD and SZ, none of whom had a diagnosis of psychosis; hence why we decided to focus on prodromal symptoms. The correlation between intrinsic connectivity and symptomatology may thus emerge as the sample grows older and approaches the peak age of onset of the disease; the association between neural signal and clinical symptoms being too subtle to be detected at this early stage. On the other hand, we observed a positive correlation between BGN connectivity signal and GMV in the left caudate, restricted to SZ-offspring, indicating that lower connectivity is associated with smaller GMV in this region in SZ-offspring, despite the absence of case-control differences in volume. In a sample of adolescent SZ-offspring, Tandon and colleagues observed reductions in N-acetyl aspartate in the caudate, thalamus and anterior cingulate gyrus and increases in glutamate + glutamine in the caudate and thalamus, which correlated with respective GMV [56]. The authors postulated that these findings could reflect abnormal glutamatergic–dopaminergic interactions in these regions, linking with the large body of research supporting abnormal striatal dopamine transmission in unaffected adult relatives of patients with SZ [57] and adults at clinical high risk for psychosis [58].

We did not observe differences in BGN connectivity between BD-offspring and CC-offspring. Khadka and colleagues, in an adult sample, also demonstrated that decreased striatal connectivity was specific to relatives of SZ, although they observed disruption in connectivity of the thalamus in both SZ and BD relatives [11]. Longitudinal follow-up of our sample will allow to confirm whether such abnormalities emerge with increasing age, or whether they remain unrelated to familial risk.

We also found evidence indicative of greater connectivity in BD-offspring in the right ECN, which was more prominent in their comparison to SZ-offspring. The ECN network overlaps with regions frequently activated during executive function tasks such as working memory [59]. A study in young SZ-offspring [60] found reduced fronto-parietal activation during a working memory task, which suggests that abnormalities in the ECN may be more reliably present when the network is actively involved in a task. However, this finding may also be reflective of compensatory network connectivity in BD-offspring. This interpretation has also been suggested by Singh and colleagues who observed increased ECN connectivity in child and adolescent BD-offspring [30]. Nevertheless, given that these results were uncorrected for multiple comparisons conclusions remain tentative.

Contrary to previous studies [27], we found no differences of connectivity in the DMN. Of note, our DMN component did not include the medial prefrontal cortex, which we hypothesize may be due to the young age of the sample. The age-related re-organization of the DMN involves increased functional connectivity, particularly in the medial prefrontal cortex [61]. Abnormalities related to risk of SZ and BD in this network may thus emerge as this sample grows into adulthood.

The SN did not yield significant results in any of the contrasts studied. Some functional alterations have been previously reported in patients with SZ [15], and abnormal structural covariance within this network has been reported in prodromal subjects who later transitioned to psychosis [62]. It is therefore possible that such dysfunction may be specific to those individuals who will later develop psychosis, and therefore not detectable at the group level. Furthermore, some studies have considered subcortical structures as a part of this network [15, 62], while in our study we have differentiated between cortico-subcortical and a fronto-insular network, which may have contributed to the lack of results in our salience component.

This study has a number of limitations. Given its cross-sectional design, we are unable to comment on the development of RSN in high-risk offspring. We included offspring receiving psychotropic medication or with different diagnoses in order to capture the clinical complexity of high-risk samples [63], although this did not influence the results. The main strength of our study is the direct comparison of SZ-offspring and BD-offspring during youth, which coincides with the age at which the incidence of both disorders begins to rise [64]. It has been suggested that research focused on adult samples may be missing important ethiopathogenic processes which take place earlier during development [65].

In conclusion, our findings point to a measurable pattern of brain dysconnectivity across RSN in child and adolescent offspring of SZ, while no functional connectivity differences were observed in BD-offspring relative to CC-offspring. Follow-up of the current sample will allow tracking whether the current changes are predictive of transition to psychosis.

## References

[1] Chai XJ, Whitfield-Gabrieli S, Shinn AK, Gabrieli JD, Nieto Castañón A, McCarthy JM, et al Abnormal medial prefrontal cortex resting-state connectivity in bipolar disorder and schizophrenia. Neuropsychopharmacology. 2011 9;36(10):2009–2017. 10.1038/npp.2011.88 21654735 PMC3158318

[2] Lichtenstein P, Yip BH, Björk C, Pawitan Y, Cannon TD, Sullivan PF, et al Common genetic determinants of schizophrenia and bipolar disorder in Swedish families: a population-based study. Lancet. 2009 1 17;373(9659):234–239. 10.1016/S0140-6736(09)60072-6 19150704 PMC3879718

[3] Keshavan MS, Morris DW, Sweeney JA, Pearlson G, Thaker G, Seidman LJ, et al A dimensional approach to the psychosis spectrum between bipolar disorder and schizophrenia: the Schizo-Bipolar Scale. Schizophr Res. 2011 12;133(1–3):250–254. 10.1016/j.schres.2011.09.005 21996268 PMC3381911

[4] Bora E, Pantelis C. Meta-analysis of Cognitive Impairment in First-Episode Bipolar Disorder: Comparison With First-Episode Schizophrenia and Healthy Controls. Schizophr Bull. 2015 9;41(5):1095–1104. 10.1093/schbul/sbu198 25616505 PMC4535631

[5] Ellison-Wright I, Bullmore E. Anatomy of bipolar disorder and schizophrenia: a meta-analysis. Schizophr Res. 2010 3;117(1):1–12. 10.1016/j.schres.2009.12.022 20071149

[6] Delvecchio G, Sugranyes G, Frangou S. Evidence of diagnostic specificity in the neural correlates of facial affect processing in bipolar disorder and schizophrenia: a meta-analysis of functional imaging studies. Psychol Med. 2013 3;43(3):553–569. 10.1017/S0033291712001432 22874625

[7] Frangou S. A systems neuroscience perspective of schizophrenia and bipolar disorder. Schizophr Bull. 2014 5;40(3):523–531. 10.1093/schbul/sbu017 24609453 PMC3984528

[8] Biswal BB, Van Kylen J, Hyde JS. Simultaneous assessment of flow and BOLD signals in resting-state functional connectivity maps. NMR Biomed. 1997 Jun-Aug;10(4–5):165–170. 9430343 10.1002/(sici)1099-1492(199706/08)10:4/5<165::aid-nbm454>3.0.co;2-7

[9] Fornito A, Harrison BJ. Brain connectivity and mental illness. Front Psychiatry. 2012 7 27;3:72 10.3389/fpsyt.2012.00072 22866039 PMC3406306

[10] Strakowski SM, Adler CM, Almeida J, Altshuler LL, Blumberg HP, Chang KD, et al The functional neuroanatomy of bipolar disorder: a consensus model. Bipolar Disord. 2012 6;14(4):313–325. 10.1111/j.1399-5618.2012.01022.x 22631617 PMC3874804

[11] Khadka S, Meda SA, Stevens MC, Glahn DC, Calhoun VD, Sweeney JA, et al Is aberrant functional connectivity a psychosis endophenotype? A resting state functional magnetic resonance imaging study. Biol Psychiatry. 2013 9 15;74(6):458–466. 10.1016/j.biopsych.2013.04.024 23746539 PMC3752322

[12] Baker JT, Holmes AJ, Masters GA, Yeo BT, Krienen F, Buckner RL, et al Disruption of cortical association networks in schizophrenia and psychotic bipolar disorder. JAMA Psychiatry. 2014 2;71(2):109–118. 10.1001/jamapsychiatry.2013.3469 24306091 PMC4435541

[13] Buckner RL, Andrews-Hanna JR, Schacter DL. The brain's default network: anatomy, function, and relevance to disease. Ann N Y Acad Sci. 2008 3;1124:1–38. 10.1196/annals.1440.011 18400922

[14] Raichle ME, MacLeod AM, Snyder AZ, Powers WJ, Gusnard DA, Shulman GL. A default mode of brain function. Proc Natl Acad Sci U S A. 2001 1 16;98(2):676–682. 11209064 10.1073/pnas.98.2.676 PMC14647

[15] Orliac F, Naveau M, Joliot M, Delcroix N, Razafimandimby A, Brazo P, et al Links among resting-state default-mode network, salience network, and symptomatology in schizophrenia. Schizophr Res. 2013 8;148(1–3):74–80. 10.1016/j.schres.2013.05.007 23727217

[16] Tang J, Liao Y, Song M, Gao JH, Zhou B, Tan C, et al Aberrant default mode functional connectivity in early onset schizophrenia. PLoS One. 2013 7 29;8(7):e71061 10.1371/journal.pone.0071061 23923052 PMC3726582

[17] Lois G, Linke J, Wessa M. Altered functional connectivity between emotional and cognitive resting state networks in euthymic bipolar I disorder patients. PLoS One. 2014 10 24;9(10):e107829 10.1371/journal.pone.0107829 eCollection 2014. 25343370 PMC4208743

[18] Magioncalda P, Martino M, Conio B, Escelsior A, Piaggio N, Presta A, et al Functional connectivity and neuronal variability of resting state activity in bipolar disorder—reduction and decoupling in anterior cortical midline structures. Hum Brain Mapp. 2015 2;36(2):666–682. 10.1002/hbm.22655 25307723 PMC6869107

[19] Seeley WW, Menon V, Schatzberg AF, Keller J, Glover GH, Kenna H, et al Dissociable intrinsic connectivity networks for salience processing and executive control. J Neurosci. 2007 2 28;27(9):2349–2356. 17329432 10.1523/JNEUROSCI.5587-06.2007 PMC2680293

[20] Menon V. Large-scale brain networks and psychopathology: a unifying triple network model. Trends Cogn Sci. 2011 10;15(10):483–506. 10.1016/j.tics.2011.08.003 21908230

[21] Tu PC, Lee YC, Chen YS, Li CT, Su TP. Schizophrenia and the brain's control network: aberrant within- and between-network connectivity of the frontoparietal network in schizophrenia. Schizophr Res. 2013 7;147(2–3):339–347. 10.1016/j.schres.2013.04.011 23706416

[22] Wu M, Lu LH, Passarotti AM, Wegbreit E, Fitzgerald J, Pavuluri MN (2013). Altered affective, executive and sensorimotor resting state networks in patients with pediatric mania. *Journal of Psychiatry & Neuroscience* 38, 232–240. 23735583 10.1503/jpn.120073 PMC3692720

[23] Argyelan M, Ikuta T, DeRosse P, Braga RJ, Burdick KE, John M, Kingsley PB, et al Resting-state fMRI connectivity impairment in schizophrenia and bipolar disorder. Schizophr Bull. 2014 1;40(1):100–110. 10.1093/schbul/sbt092 23851068 PMC3885301

[24] Lui S, Yao L, Xiao Y, Keedy SK, Reilly JL, Keefe RS, et al Resting-state brain function in schizophrenia and psychotic bipolar probands and their first-degree relatives. Psychol Med. 2015 1;45(1):97–108. 10.1017/S003329171400110X 25066779 PMC5836742

[25] Jang JH, Jung WH, Choi JS, Choi CH, Kang DH, Shin NY, et al Reduced prefrontal functional connectivity in the default mode network is related to greater psychopathology in subjects with high genetic loading for schizophrenia. Schizophr Res. 2011 4;127(1–3):58–65. 10.1016/j.schres.2010.12.022 21277171

[26] van Buuren M, Vink M, Kahn RS. Default-mode network dysfunction and self-referential processing in healthy siblings of schizophrenia patients. Schizophr Res. 2012 12;142(1–3):237–243. 10.1016/j.schres.2012.09.017 23099059

[27] Jukuri T, Kiviniemi V, Nikkinen J, Miettunen J, Mäki P, Jääskeläinen E, et al Default mode network in young people with familial risk for psychosis—the Oulu Brain and Mind study. Schizophr Res. 2013 2;143(2–3):239–245. 10.1016/j.schres.2012.11.020 23245776

[28] Meda SA, Gill A, Stevens MC, Lorenzoni RP, Glahn DC, Calhoun VD, et al Differences in resting-state functional magnetic resonance imaging functional network connectivity between schizophrenia and psychotic bipolar probands and their unaffected first-degree relatives. Biol Psychiatry. 2012 5 15;71(10):881–889. 10.1016/j.biopsych.2012.01.025 22401986 PMC3968680

[29] Jukuri T, Kiviniemi V, Nikkinen J, Miettunen J, Mäki P, Mukkala S, et al Central executive network in young people with familial risk for psychosis—the Oulu Brain and Mind Study. Schizophr Res. 2015 2;161(2–3):177–183. 10.1016/j.schres.2014.11.003 25468181

[30] Singh MK, Chang KD, Kelley RG, Saggar M, Reiss AL, Gotlib IH. Early signs of anomalous neural functional connectivity in healthy offspring of parents with bipolar disorder. Bipolar Disord. 2014 11;16(7):678–689. 10.1111/bdi.12221 24938878 PMC4213354

[31] Fornito A, Harrison BJ, Goodby E, Dean A, Ooi C, Nathan PJ, et al Functional dysconnectivity of corticostriatal circuitry as a risk phenotype for psychosis. JAMA Psychiatry. 2013 11;70(11):1143–1151. 10.1001/jamapsychiatry.2013.1976 24005188

[32] Power JD, Fair DA, Schlaggar BL, Petersen SE. The development of human functional brain networks. Neuron. 2010 9 9;67(5):735–748. 10.1016/j.neuron.2010.08.017 20826306 PMC2941973

[33] Supekar K, Uddin LQ, Prater K, Amin H, Greicius MD, Menon V. Development of functional and structural connectivity within the default mode network in young children. Neuroimage. 2010 8 1;52(1):290–301. 10.1016/j.neuroimage.2010.04.009 20385244 PMC2976600

[34] Ordaz SJ, Foran W, Velanova K, Luna B. Longitudinal growth curves of brain function underlying inhibitory control through adolescence. J Neurosci. 2013 11 13;33(46):18109–18124. 10.1523/JNEUROSCI.1741-13.2013 24227721 PMC3828464

[35] Porter JN, Roy AK, Benson B, Carlisi C, Collins PF, Leibenluft E, et al Age-related changes in the intrinsic functional connectivity of the human ventral vs. dorsal striatum from childhood to middle age. Dev Cogn Neurosci. 2015 2;11:83–95. 10.1016/j.dcn.2014.08.011 25257972 PMC6310902

[36] Maziade M, Rouleau N, Mérette C, Cellard C, Battaglia M, Marino C, et al Verbal and visual memory impairments among young offspring and healthy adult relatives of patients with schizophrenia and bipolar disorder: selective generational patterns indicate different developmental trajectories. Schizophr Bull. 2011 11;37(6):1218–28. 10.1093/schbul/sbq026 20410238 PMC3196959

[37] Diwadkar VA, Goradia D, Hosanagar A, Mermon D, Montrose DM, Birmaher B, et al Working memory and attention deficits in adolescent offspring of schizophrenia or bipolar patients: comparing vulnerability markers. Prog Neuropsychopharmacol Biol Psychiatry. 2011 7 1;35(5):1349–54. 10.1016/j.pnpbp.2011.04.009 21549798 PMC3126676

[38] American Psychiatric Association (APA). Diagnostic and Statistical Manual of Mental Disorders (DSM-IV); American Psychiatric Association; Washington, DC 1994.

[39] Sanchez-Gistau V, Romero S, Moreno D, de la Serna E, Baeza I, Sugranyes G, et al Psychiatric disorders in child and adolescent offspring of patients with schizophrenia and bipolar disorder: A controlled study. Schizophr Res. 2015 10;168(1–2):197–203. 10.1016/j.schres.2015.08.034 26363969

[40] Hollingshead AB, Redlich FC. Social Class and Mental Illness. Wiley: New York; 1958. 10.2105/ajph.97.10.1756 PMC1994199 17895405

[41] First MB, Spitzer RL, Gibbon M, Williams JBW. Structured Clinical Interview for DSM-IV-TR Axis I Disorders, Research Version, Patient Edition (SCID-I/P) New York: Biometrics Research, New York State Psychiatric Institute; 2002.

[42] Geller B, Zimerman B, Williams M, Bolhofner K, Craney JL, DelBello MP, et al Reliability of the Washington University in St. Louis Kiddie Schedule for Affective Disorders and Schizophrenia (WASH-U-KSADS) mania and rapid cycling sections. J Am Acad Child Adolesc Psychiatry. 2001 4;40(4):450–455. 11314571 10.1097/00004583-200104000-00014

[43] Miller TJ, McGlashan TH, Rosen JL, Cadenhead K, Cannon T, Ventura J, et al Prodromal assessment with the structured interview for prodromal syndromes and the scale of prodromal symptoms: predictive validity, interrater reliability, and training to reliability. Schizophr Bull. 2003;29(4):703–715. 14989408 10.1093/oxfordjournals.schbul.a007040

[44] Young RC, Biggs JT, Ziegler VE, Meyer DA (1978). A rating scale for mania: reliability, validity and sensitivity. The *British Journal of Psychiatry* 133, 429–435. 728692 10.1192/bjp.133.5.429

[45] Hamilton M. Development of a rating scale for primary depressive illness. Br J Soc Clin Psychol. 1967 12;6(4):278–296. 6080235 10.1111/j.2044-8260.1967.tb00530.x

[46] Busner J, Targum SD. The clinical global impressions scale: applying a research tool in clinical practice. Psychiatry (Edgmont). 2007 7;4(7):28–37. PMC2880930 20526405

[47] Wechsler D. The Wechsler intelligence scale for children III. Paidós: Buenos Aires; 1994.

[48] Wechsler D. Wechlser Adult Intelligence Scale III. TEA ediciones: Madrid; 1999.

[49] Burgund ED, Kang HC, Kelly JE, Buckner RL, Snyder AZ, Petersen SE, et al The feasibility of a common stereotactic space for children and adults in fMRI studies of development. Neuroimage. 2002 9;17(1):184–200. 12482076 10.1006/nimg.2002.1174

[50] Erhardt EB, Rachakonda S, Bedrick EJ, Allen EA, Adali T, Calhoun VD. Comparison of multi-subject ICA methods for analysis of fMRI data. Hum Brain Mapp. 2011 12;32(12): 2075–2095. 10.1002/hbm.21170 21162045 PMC3117074

[51] Shirer WR, Ryali S, Rykhlevskaia E, Menon V, Greicius MD. Decoding subject-driven cognitive states with whole-brain connectivity patterns. Cereb Cortex. 2012 1;22(1):158–165. 10.1093/cercor/bhr099 21616982 PMC3236795

[52] Taki Y, Thyreau B, Kinomura S, Sato K, Goto R, Kawashima R et al Correlations among brain gray matter volumes, age, gender, and hemisphere in healthy individuals. PLoS One. 2011;6(7):e22734 10.1371/journal.pone.0022734 21818377 PMC3144937

[53] Fischl B, Sereno MI, Dale AM. Cortical surface-based analysis. II: Inflation, flattening, and a surface-based coordinate system. Neuroimage. 1999 2;9(2):195–207. 9931269 10.1006/nimg.1998.0396

[54] Nakao T, Radua J, Rubia K, Mataix-Cols D. Gray matter volume abnormalities in ADHD: voxel-based meta-analysis exploring the effects of age and stimulant medication. Am J Psychiatry. 2011 11;168(11):1154–1163. 10.1176/appi.ajp.2011.11020281 21865529

[55] Farr OM, Zhang S, Hu S, Matuskey D, Abdelghany O, Malison RT, et al The effects of methylphenidate on resting-state striatal, thalamic and global functional connectivity in healthy adults. Int J Neuropsychopharmacol. 2014 8;17(8):1177–1191. 10.1017/S1461145714000674 24825078 PMC4506752

[56] Tandon N, Bolo NR, Sanghavi K, Mathew IT, Francis AN, Stanley JA, et al Brain metabolite alterations in young adults at familial high risk for schizophrenia using proton magnetic resonance spectroscopy. Schizophr Res. 2013 8;148(1–3):59–66. 10.1016/j.schres.2013.05.024 23791389

[57] Huttunen J, Heinimaa M, Svirskis T, Nyman M, Kajander J, Forsback S, et al Striatal dopamine synthesis in first-degree relatives of patients with schizophrenia. Biol Psychiatry. 2008 1 1;63(1):114–117. 17655830 10.1016/j.biopsych.2007.04.017

[58] Fusar-Poli P, Howes OD, Allen P, Broome M, Valli I, Asselin MC, et al Abnormal prefrontal activation directly related to pre-synaptic striatal dopamine dysfunction in people at clinical high risk for psychosis. Mol Psychiatry. 2011 1;16(1):67–75. 10.1038/mp.2009.108 19949389

[59] Laird AR, Fox PM, Eickhoff SB, Turner JA, Ray KL, McKay DR, et al Behavioral interpretations of intrinsic connectivity networks. J Cogn Neurosci. 2011 12;23(12):4022–4037. 10.1162/jocn_a_00077 21671731 PMC3690655

[60] Diwadkar VA, Pruitt P, Goradia D, Murphy E, Bakshi N, Keshavan MS, et al Fronto-parietal hypo-activation during working memory independent of structural abnormalities: conjoint fMRI and sMRI analyses in adolescent offspring of schizophrenia patients. Neuroimage. 2011 9 1;58(1):234–241. 10.1016/j.neuroimage.2011.06.033 21729757 PMC3164159

[61] Sato JR, Salum GA, Gadelha A, Picon FA, Pan PM, Vieira G, et al Age effects on the default mode and control networks in typically developing children. J Psychiatr Res. 2014 11;58:89–95. 10.1016/j.jpsychires.2014.07.004 25085608

[62] Heinze K, Reniers RL, Nelson B, Yung AR, Lin A, Harrison BJ, et al Discrete alterations of brain network structural covariance in individuals at ultra-high risk for psychosis. Biol Psychiatry. 2015 6 1;77(11):989–996. 10.1016/j.biopsych.2014.10.023 25524754

[63] Rasic D, Hajek T, Alda M, Uher R. Risk of mental illness in offspring of parents with schizophrenia, bipolar disorder, and major depressive disorder: a meta-analysis of family high-risk studies. Schizophr Bull. 2014 1;40(1):28–38. 10.1093/schbul/sbt114 23960245 PMC3885302

[64] Jones PB. Adult mental health disorders and their age at onset. Br J Psychiatry Suppl. 2013 1;54:s5–10. 10.1192/bjp.bp.112.119164 23288502

[65] Insel TR. Rethinking schizophrenia. Nature. 2010 11 11;468(7321):187–93. 10.1038/nature09552 21068826

